# RNA-Seq analysis of *Gtf2ird1* knockout epidermal tissue provides potential insights into molecular mechanisms underpinning Williams-Beuren syndrome

**DOI:** 10.1186/s12864-016-2801-4

**Published:** 2016-06-13

**Authors:** Susan M. Corley, Cesar P. Canales, Paulina Carmona-Mora, Veronica Mendoza-Reinosa, Annemiek Beverdam, Edna C. Hardeman, Marc R. Wilkins, Stephen J. Palmer

**Affiliations:** Systems Biology Initiative, School of Biotechnology and Biomolecular Sciences, UNSW Australia, Sydney, NSW Australia; Cellular and Genetic Medicine Unit, School of Medical Sciences, UNSW Australia, Sydney, NSW Australia; School of Medical Sciences, UNSW Australia, Sydney, NSW Australia

**Keywords:** RNA-Seq, Transcriptomics, Williams-Beuren syndrome, Neurodevelopmental disorder

## Abstract

**Background:**

Williams-Beuren Syndrome (WBS) is a genetic disorder associated with multisystemic abnormalities, including craniofacial dysmorphology and cognitive defects. It is caused by a hemizygous microdeletion involving up to 28 genes in chromosome 7q11.23. Genotype/phenotype analysis of atypical microdeletions implicates two evolutionary-related transcription factors, GTF2I and GTF2IRD1, as prime candidates for the cause of the facial dysmorphology.

**Results:**

Using a targeted *Gtf2ird1* knockout mouse, we employed massively-parallel sequencing of mRNA (RNA-Seq) to understand changes in the transcriptional landscape associated with inactivation of *Gtf2ird1* in lip tissue. We found widespread dysregulation of genes including differential expression of 78 transcription factors or coactivators, several involved in organ development including *Hey1, Myf6, Myog, Dlx2, Gli1, Gli2, Lhx2, Pou3f3, Sox2, Foxp3*. We also found that the absence of GTF2IRD1 is associated with increased expression of genes involved in cellular proliferation, including growth factors consistent with the observed phenotype of extreme thickening of the epidermis. At the same time, there was a decrease in the expression of genes involved in other signalling mechanisms, including the Wnt pathway, indicating dysregulation in the complex networks necessary for epidermal differentiation and facial skin patterning. Several of the differentially expressed genes have known roles in both tissue development and neurological function, such as the transcription factor *Lhx2* which regulates several genes involved in both skin and brain development.

**Conclusions:**

*Gtf2ird1* inactivation results in widespread gene dysregulation, some of which may be due to the secondary consequences of gene regulatory network disruptions involving several transcription factors and signalling molecules. Genes involved in growth factor signalling and cell cycle progression were identified as particularly important for explaining the skin dysmorphology observed in this mouse model. We have noted that a number of the dysregulated genes have known roles in brain development as well as epidermal differentiation and maintenance. Therefore, this study provides clues as to the underlying mechanisms that may be involved in the broader profile of WBS.

**Electronic supplementary material:**

The online version of this article (doi:10.1186/s12864-016-2801-4) contains supplementary material, which is available to authorized users.

## Background

*Gtf2ird1* is a member of the *Gtf2i* family of genes, encoding a set of multifunctional transcription factors. The three members of this family cluster within a domain of the 7q11.23 chromosomal region that is prone to copy number variation through non-allelic homologous recombination. Hemizygous deletion of this domain leads to the neurodevelopmental disorder, Williams-Beuren Syndrome (WBS) [1]. This is a multisystem disorder with physical, cognitive and behavioural components. Studies of WBS patients with atypical deletions of the region have led to the conclusion that loss of *GTF2IRD1* and *GTF2I* explain prominent features of the condition such as the craniofacial dysmorphology, the intellectual disability and the behavioural effects [2].

Analysis of mouse knockouts of the orthologous genes *Gtf2ird1* and *Gtf2i* support these conclusions. Homozygous loss of *Gtf2i* causes embryonic lethality [3] but heterozygous loss results in increased anxiety, as measured by the light–dark box and elevated plus maze tests [4]. Homozygous *Gtf2ird1* mutants show reduced levels of aggression in the resident intruder test [5] impaired motor coordination and exploratory behaviour [6, 7] and altered vocalization in response to specific stress-inducing cues [6]. Homozygous loss of *Gtf2ird1* has also been shown to cause craniofacial abnormalities that in a transgene insertion mutant, affects the alignment of the jaws [8], but in the knockout model presented in this work, is mainly confined to an excessive overgrowth of the soft tissue [6]. This phenotype is not apparent in the heterozygous *Gtf2ird1* knockout mice. Detailed analysis of adult homozygous null *Gtf2ird1*^*tm1Hrd*^ mice demonstrated that these abnormalities were confined to the regions around the lips and the nose and histological sections showed that the increased thickness was due to an enlargement of the epidermal layer that sometimes resulted in skin flaps and folds in the lips and around the nares [6]. While no histology of the face is available from WBS patients, it has been noted that the majority of the WBS craniofacial phenotype is soft tissue related [6].

*Gtf2ird1* expression in the mouse is consistent with a role in craniofacial development and brain function [9]. The *Gtf2ird1* transcript is detectable from early stages of embryogenesis through to the development of specific tissues including cartilage, muscle, heart, brain and tooth buds. In the developing head, many of the hard and soft tissue components express *Gtf2ird1*. In the transition to maturity, many of these sites are shut down and expression in the adult becomes mainly confined to sensory organs, neurons of the peripheral and central nervous system, smooth muscle, cardiac muscle, cells in the testis and brown adipose tissue [9, 10].

Several studies illustrate GTF2IRD1’s DNA binding properties [11, 12] and its ability to act as a transcriptional regulator in transgenic mouse systems [13], as well as a capacity to auto-regulate its own transcription through direct binding of the GTF2IRD1 protein to its own promoter region [14]. However, much of the information regarding GTF2IRD1 gene targets remains unknown. Analyses of *Gtf2ird1* knockout brain tissue, for example, have led to a disappointing lack of useful information [15]. Other studies have examined gene expression differences in mouse embryonic fibroblasts (MEFs) that overexpress *Gtf2ird1* [16, 17] and in a *Gtf2ird1* gene-trap mutant mouse model [3] that shows phenotypic defects that are more extreme than the *Gtf2ird1* deletion models [5, 6, 8, 14]. This is the first published report of a comprehensive RNA-Seq analysis of the transcriptome of mice deficient for *Gtf2ird1*.

In this study we have analysed lip tissue from a *Gtf2ird1* knockout mouse model in order to capture effects that are most apparent in the epidermis. Our observation that certain dysregulated genes have roles both in skin and brain development suggests that this analysis can provide insight into molecular effectors and pathways involved in WBS.

## Results

### RNA-Seq analysis shows widespread gene dysregulation in the *Gtf2ird1* KO

RNA was extracted from mouse lip tissue of three female *Gtf2ird1* knockout mice (KO) and three female wild type mice (WT). The KO mice had a distinct physical appearance with overgrowth and wrinkling of the lip tissue, consistent with the phenotype previously described [6]. The six RNA-Seq libraries were sequenced on the Illumina HiSeq2000 platform to produce over 60 million, 100 nucleotide paired-end reads per sample. Differential expression analysis was performed using the Bioconductor packages, edgeR [18] and subsequently confirmed using DESeq2 [19], as detailed in Methods. In this paper, we describe the RNA-Seq results broadly and identify differentially expressed genes with roles in normal epidermal and neurological development.

### Differential gene expression seen in both directions in the *Gtf2ird1* KO

**Fig. 1:**
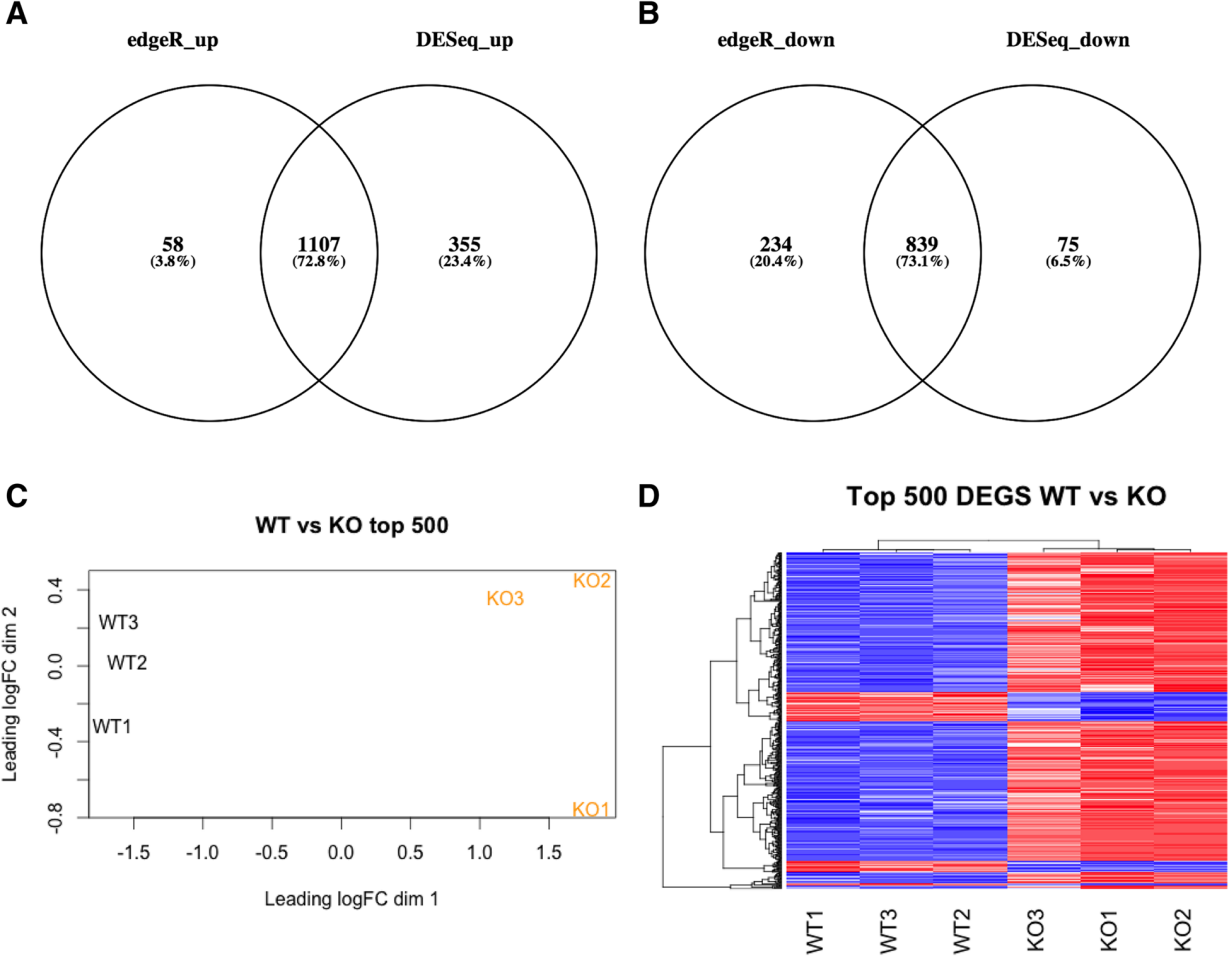
Differential expression analysis. **a, b** Venn diagrams of the sets of differentially expressed genes (DEGs) generated using different methods for analyzing differential expression. **a** Genes up-regulated in KO, and (b) genes down-regulated in KO found using edgeR and DESeq2. **c** Multidimensional scaling (MDS) plot generated using edgeR showing clustering of samples with the 3 biological replicates of WT (black) and 3 biological replicates of KO (orange). **d** Heatmap generated using heatmap.2 function of the gplots package in R of the top 500 differentially expressed genes found using edgeR, with input being the logCPM values for these genes. Blue represents lower expression, red represents higher expression

Differential expression analysis of the KO and WT lip tissue with edgeR identified 1165 genes with significantly increased expression and 1073 genes with significantly decreased expression (edgeR, FDR = 0.05) (Fig. 1a, b). We found a large overlap in genes called as differentially expressed using edgeR [18] and DESeq2 [19] of all the genes found by either method, 73 % were common to both methods (Fig. 1a, b). The WT and KO conditions could be clearly distinguished using the multidimensional scaling (MDS) plot (Fig. 1c). The clear separation of the two genotypes was also evident in the heatmap showing the expression level of the top 500 differentially expressed genes (Fig. 1d). Although a similar total number of up-regulated and down-regulated genes were identified, when ordered by statistical significance (lowest FDR values) we found that the most statistically significant differentially expressed genes in the KO are up-regulated as depicted in the heatmap (Fig. 1d). The lists of up-regulated and down-regulated genes found using edgeR were sorted by statistical significance (lowest FDR) and the top 50 in the up-regulated and down-regulated groups are listed in Table 1. Details of the differentially expressed genes can be found in Additional file 1: Table S1.

**Table 1:** Top 50 up-regulated and down-regulated genes found using edgeR

Up-regulated genes	logFC^a^	FDR^b^	Down-regulated genes	logFC^a^	FDR^b^
Slc14a2	8.39	5.0E-117	Krt2	−4.52	1.7E-57
Gm16026	10.57	8.7E-111	Acsm1	−3.35	8.8E-13
Lrrn4	9.33	8.7E-111	1100001G20Rik	−2.54	1.6E-12
Cml2	9.16	4.6E-94	F3	−1.51	2.1E-12
Sp110	7.82	7.4E-90	Tac4	−1.79	7.0E-12
Gm15753	8.36	4.0E-87	Cyp2w1	−2.50	9.6E-12
Prr18	6.13	1.4E-75	Cacng1	−1.86	6.5E-10
C130026I21Rik	9.14	2.0E-75	Ces2f	−1.99	4.8E-09
Echdc3	4.48	2.0E-66	Paqr5	−1.34	6.3E-09
AI427809	5.10	6.2E-61	Cyp2f2	−2.11	6.9E-09
Dhtkd1	4.31	2.0E-58	Gm1110	−2.87	5.0E-08
Gm7029	10.86	3.0E-58	Sebox	−2.09	9.8E-08
Gm5335	9.11	3.2E-58	Wnt7b	−1.36	1.0E-07
Ccdc60	6.30	1.5E-56	Gm26888	−2.27	2.0E-07
Apol7b	7.45	3.1E-55	Slc6a19	−1.26	2.5E-07
0610040J01Rik	4.75	5.3E-53	Ucp3	−1.67	5.0E-07
Ccser1	5.02	2.7E-52	Capn9	−2.16	9.9E-07
Gm8674	9.03	9.9E-51	Actc1	−1.74	1.5E-06
Fbxo16	5.43	1.6E-50	H19	−1.39	1.5E-06
BB557941	8.95	2.5E-50	Lypd6	−1.48	1.6E-06
Gm12114	7.01	3.2E-50	Slc46a1	−1.22	3.5E-06
Gm13191	8.71	4.6E-50	Hsd17b14	−1.35	5.6E-06
Tpo	7.68	4.6E-50	Csrp3	−1.21	6.7E-06
Gm16239	7.43	3.0E-49	Gm1078	−2.06	7.2E-06
Kcnk15	7.19	3.5E-49	Awat1	−1.09	1.4E-05
Chrm3	4.67	5.0E-49	Gm10228	−1.47	1.4E-05
Mecom	4.37	7.2E-49	Plin5	−1.57	1.5E-05
Gm7592	3.94	5.4E-48	Pdk4	−2.07	1.6E-05
Tie1	3.54	8.1E-48	Fam57b	−1.32	1.6E-05
C86695	10.24	1.5E-46	Gfra2	−1.23	1.7E-05
Dlgap2	7.01	2.9E-46	Mybph	−1.42	2.6E-05
Colgalt2	4.66	4.1E-46	Myoz2	−1.43	3.4E-05
Ush2a	7.52	2.4E-45	Fabp3	−1.13	3.4E-05
A530040E14Rik	6.04	1.0E-44	Tfr2	−1.74	3.5E-05
B3gntl1	3.71	4.4E-44	Zbtb16	−1.80	3.6E-05
Gm10653	5.36	5.3E-44	Skint10	−1.52	3.9E-05
Gm12495	8.97	5.5E-44	Mustn1	−1.18	4.5E-05
Dcc	8.26	3.8E-43	AU021092	−1.40	4.8E-05
AU019990	6.31	7.2E-42	Tspear	−1.53	4.9E-05
Gm16028	10.30	3.8E-41	Krtap20-2	−1.67	5.0E-05
Arhgap8	3.83	1.3E-38	Alox15	−2.47	5.3E-05
Oas1a	5.00	2.6E-38	Krt36	−0.95	5.4E-05
AI481877	6.11	3.1E-38	Rarres1	−1.10	5.7E-05
BC026585	3.29	6.6E-38	P2ry4	−1.22	7.1E-05
Sp140	4.27	5.1E-37	Cyp17a1	−1.82	7.7E-05
Aqp9	3.00	7.9E-37	Ankrd2	−1.49	8.1E-05
Abcb5	9.79	1.0E-36	Foxq1	−1.19	8.1E-05
Gm12724	6.50	1.7E-36	Vgll2	−1.62	8.6E-05
Defb13	7.65	1.9E-36	Gm12551	−1.16	8.8E-05
Ric3	4.41	3.2E-36	Pdlim3	−1.09	1.1E-04

^a^LogFC: fold change expressed as log base 2

^b^FDR: *p* value adjusted using Benjamini Hochberg method implemented in edgeR

The genes listed in Table 1 have functions in transcription regulation (*Foxq1, Sebox, Csrp3, Vgll2, Zbtb16, Sp110*), signalling (*Wnt7, Chrm3, P2ry4, Arhgap8, Au021092, Lypd6, F3, Skint10, Tac4, Gfra2, Dcc, Lrrn4, Defb13, Tie1, Ush2a*), calcium binding (*Pdlim3, Acsm1, Alox15, Cacng1, Capn9, Csrp3, Cyp2f2, Gm1110, Zbtb16*), membrane transport (*Slc6a19, Slc46a1, Aqp9*), apoptosis (*Csrp3, Zbtb16, Sp110, Dcc, Actc1*), neurological processes (*Chrm3, Dcc, Gfra2, Slc6a19, Ush2a*) and development and maintenance of the stratified epidermis (*Aqp9, Krt2*).

### Several transcription factors identified in the differentially expressed genes

Of the genes dysregulated by the inactivation of *Gtf2ird1*, 79 are transcription factors or cofactors (Table 2), identified using MetaCore™. Gene ontology analysis of this group of genes performed using BiNGO reveals involvement in organ/tissue development (46/79), embryonic development (29/79), cell differentiation (39/79), regulation of cell proliferation (14/79) and signalling through a number of different pathways (26/79). Full details of the gene ontology analysis of the differentially expressed transcription factors is included in Additional file 2: Table S2. The transcription factors involved in epithelium development are *Sox2*, *Mecom*, *Lmo4*, *Lhx2*, *Six1*, *Six2*, *Twist1*, *Id3*, *Pitx2*, *Tbx3*, *Gli2*. A subset of these (*Sox2*, *Mecom*, *Lhx2*, *Pitx2*, *Tbx3* and *Gli2*) are also known to be involved in brain development as are the differentially expressed transcription factors *Dlx2*, *Foxc1*, *Lef1*, *Gli1, Pou3f3, Lmx1a, Six3, Lmx1b, Msx, Pitx3 and Lhx8*. Abnormalities in the abundance of these transcription factors, caused by transcriptional dysregulation, are likely to affect the expression of their target genes. This may explain the large number of differentially expressed genes identified in this study.

**Table 2:** Differentially expressed transcription factors

Gene	logFC^a^	FDR^b^	Gene	logFC^a^	FDR^b^
Ahrr	2.34	9.3E-10	Mlxipl	−0.81	1.1E-02
Arntl2	1.23	1.1E-03	Msx1	−0.71	2.7E-02
Ascl2	−1.42	5.6E-03	Msx2	−0.92	3.1E-03
Bach2	−0.64	2.5E-02	Mycn	−0.83	2.1E-02
Bcl3	0.92	1.5E-02	Myf6	−1.27	3.0E-03
Cited2	−0.61	3.4E-02	Myog	−1.11	1.7E-02
Clock	1.18	4.1E-04	Nfe2l2	1.03	2.6E-05
Creb3l1	−0.91	2.3E-02	Nfia	−0.66	1.6E-02
Dlx2	−0.77	1.9E-02	Nfic	−0.81	3.4E-02
Ebf1	−0.71	2.4E-02	Nfil3	−0.68	3.2E-02
Ehf	1.22	2.3E-03	Patz1	−0.68	3.8E-02
Esrrb	−1.59	2.1E-02	Pax7	−1.40	1.1E-02
Esrrg	−0.79	3.1E-02	Pitx2	−0.67	2.6E-02
Fosl1	1.38	6.9E-06	Pitx3	−0.87	2.8E-02
Foxc1	−0.70	2.4E-02	Pou3f3	−1.58	5.1E-03
Foxe1	−0.94	2.5E-02	Rorc	−1.26	1.1E-04
Foxl2	−1.21	2.2E-02	Scml2	1.28	7.5E-05
Foxp3	1.43	1.6E-03	Six1	−1.13	7.5E-03
Foxq1	−1.19	8.1E-05	Six2	−1.13	2.7E-02
Gli1	−0.74	4.1E-03	Six3	3.27	1.4E-09
Gli2	−0.77	6.6E-03	Smad6	−0.80	3.0E-02
Glis1	2.73	2.0E-23	Smarca5	0.77	2.2E-02
Glis2	−0.77	1.6E-02	Sox12	−0.75	2.0E-02
Hey1	−0.81	2.8E-02	Sox15	1.19	3.7E-04
Hoxc13	−0.90	9.4E-04	Sox2	−1.08	3.0E-02
Id3	−0.65	2.2E-02	Stat1	1.81	1.9E-08
Irf7	1.98	1.6E-09	Stat2	1.60	1.1E-07
Irf9	1.84	4.0E-14	Taf1	0.90	1.4E-02
Jdp2	−0.73	4.2E-02	Tbx2	−0.94	4.7E-03
Klf2	−0.84	3.0E-02	Tbx3	−0.66	2.6E-02
Klf9	−0.75	1.8E-02	Tcf7l1	−0.75	1.8E-02
Lef1	−0.98	1.5E-03	Tead2	−0.91	2.4E-03
Lhx2	−0.72	1.8E-02	Tfcp2	1.10	2.3E-05
Lhx8	−1.00	7.6E-03	Tfeb	−0.75	2.0E-02
Lmo4	−0.73	7.5E-03	Trim25	1.02	2.8E-05
Lmx1a	4.33	1.4E-29	Tsc22d3	−1.19	1.6E-02
Lmx1b	−1.02	2.4E-02	Twist1	−0.91	2.5E-02
Maf	−0.89	7.0E-04	Zbtb16	−1.80	3.6E-05
Mafa	−1.10	1.5E-02	Zfhx3	−0.74	3.0E-02
Mecom	4.37	7.2E-49			

^a^LogFC: fold change expressed as log base 2

^b^FDR: *p* value adjusted using Benjamini Hochberg method implemented in edgeR

To test if changes in transcription factor gene expression are associated with concomitant changes in the expression of their targets, we examined *Lhx2*, a gene encoding a transcription factor important for epidermal differentiation and neurogenesis [20, 21]. We found that *Lhx2* expression is decreased by 40 % in the absence of *Gtf2ird1*. We used the MetaCore™ database (Thompson Reuters) to identify genes transcriptionally regulated by the LHX2 protein. This revealed 467 genes with known or putative binding sites for LHX2 and which are annotated as being transcriptionally regulated by *Lhx2*. We found that 363 of these were expressed in at least one of our conditions and therefore tested for differential expression. Of these, we found that 66 (18 %) are differentially regulated in this study (Table 3). Of most interest are the genes transcriptionally activated by LHX2 but which are seen in this study to have decreased expression consistent with a decrease in LHX2 such as the cluster of 10 genes involved in development (*Pdlim3*, *Mef2c*, *Aldh1a2*, *Ndn*, *Tspan12*, *Sopb*, *Thbs4*, *Ttn*, *Dact1*, *Lhx8*).

**Table 3:** Downstream targets of Lhx2 found to be differentially regulated

Gene	logFC^a^	FDR^b^	#^c^	Gene	logFC^a^	FDR^b^	#^c^
Ush2a	7.52	3.90E-46	TA	Mef2c	−0.66	4.10E-02	TA
0610040J01Rik	4.75	6.90E-54	TA	Cd34	−0.71	1.90E-02	U
Sprr2d	3.47	2.20E-04	TA	Thbs4	−0.71	3.70E-02	TA
Ceacam1	3	4.90E-27	TA	Phactr1	−0.73	2.70E-02	TA
Samd12	2.56	5.90E-08	TA	6330403K07Rik	−0.75	2.50E-02	TA
Tfec	2.29	6.60E-06	TA	Tspan12	−0.77	1.60E-02	TA
Gucy1a3	2.02	1.70E-13	TA	Loxl2	−0.79	4.40E-02	TA
Hsp90aa1	1.97	6.10E-08	TA	Aldh1a2	−0.8	8.60E-03	TA
Gbp7	1.89	1.90E-07	TA	Aldh1a2	−0.8	8.60E-03	TA
Il1f9	1.76	5.30E-08	TA	Fbxo40	−0.82	2.68E-02	TA
Gbp2	1.71	1.70E-05	TA	Htra3	−0.83	1.90E-02	TA
Robo1	1.7	2.80E-16	TI	Cxcl14	−0.84	1.70E-02	TA
Iigp1	1.69	1.60E-03	TA	Slc43a1	−0.84	1.70E-02	TA
Ccl2	1.51	7.10E-07	TI	Dact1	−0.87	3.30E-02	TA
Fam171b	1.49	2.20E-06	TA	Dysf	−0.87	4.30E-03	TA
Serpinb11	1.43	3.50E-03	TA	C1qtnf9	−0.89	2.80E-02	TA
Serpinb6c	1.36	8.50E-03	TA	Ndn	−0.89	2.70E-02	TA
Scml2	1.28	5.90E-05	TA	Sobp	−0.94	7.80E-03	TA
Slc7a11	1.2	6.14E-03	TA	Krtap13-1	−0.95	8.00E-03	TA
Dsc1	1.19	4.30E-03	TA	Krtap17-1	−0.95	3.60E-03	TA
Ifi47	1.05	4.80E-02	TA	Has1	−0.96	4.40E-02	TI
Mphosph10	1.03	8.70E-03	TI	Lhx8	−1	8.90E-03	TA
Esd	1.02	9.00E-05	TA	Ranbp17	−1.01	4.20E-02	TA
Tdrd7	0.97	5.70E-04	TA	Gnb3	−1.09	4.00E-02	TA
Parp8	0.94	1.30E-04	TI	Pdlim3	−1.09	1.00E-04	TA
Itsn2	0.9	1.50E-02	TA	Krtap8-1	−1.16	6.50E-03	TA
Lce1h	0.86	3.40E-02	TA	Ttn	−1.17	4.00E-02	TA
Casp1	0.85	4.90E-02	TA	Il22ra2	−1.19	2.10E-02	TI
Cast	0.83	1.20E-02	TA	Krtap16-3	−1.19	1.70E-03	TA
Fgf1	0.79	3.90E-03	TI	Krtap14	−1.29	1.40E-03	TA
Lce1e	0.79	4.40E-02	TA	Ankrd2	−1.49	7.50E-05	U
Plxna2	0.55	4.40E-02	TA	Acsm1	−3.35	8.10E-13	TA
Cyp2d22	−0.61	3.60E-02	TA	Chi3l4	−6.56	1.96E-03	TA

^a^LogFC: fold change expressed as log base 2

^b^FDR: *p* value adjusted using Benjamini Hochberg method implemented in edgeR

^c^Mechanism by which Lhx2 acts on target gene, TA: Transcription activator, TI: Transcription inhibitor, U: unknown

### Functional analysis of all the differentially expressed genes using gene ontology analysis and gene set enrichment

**Fig. 2:**
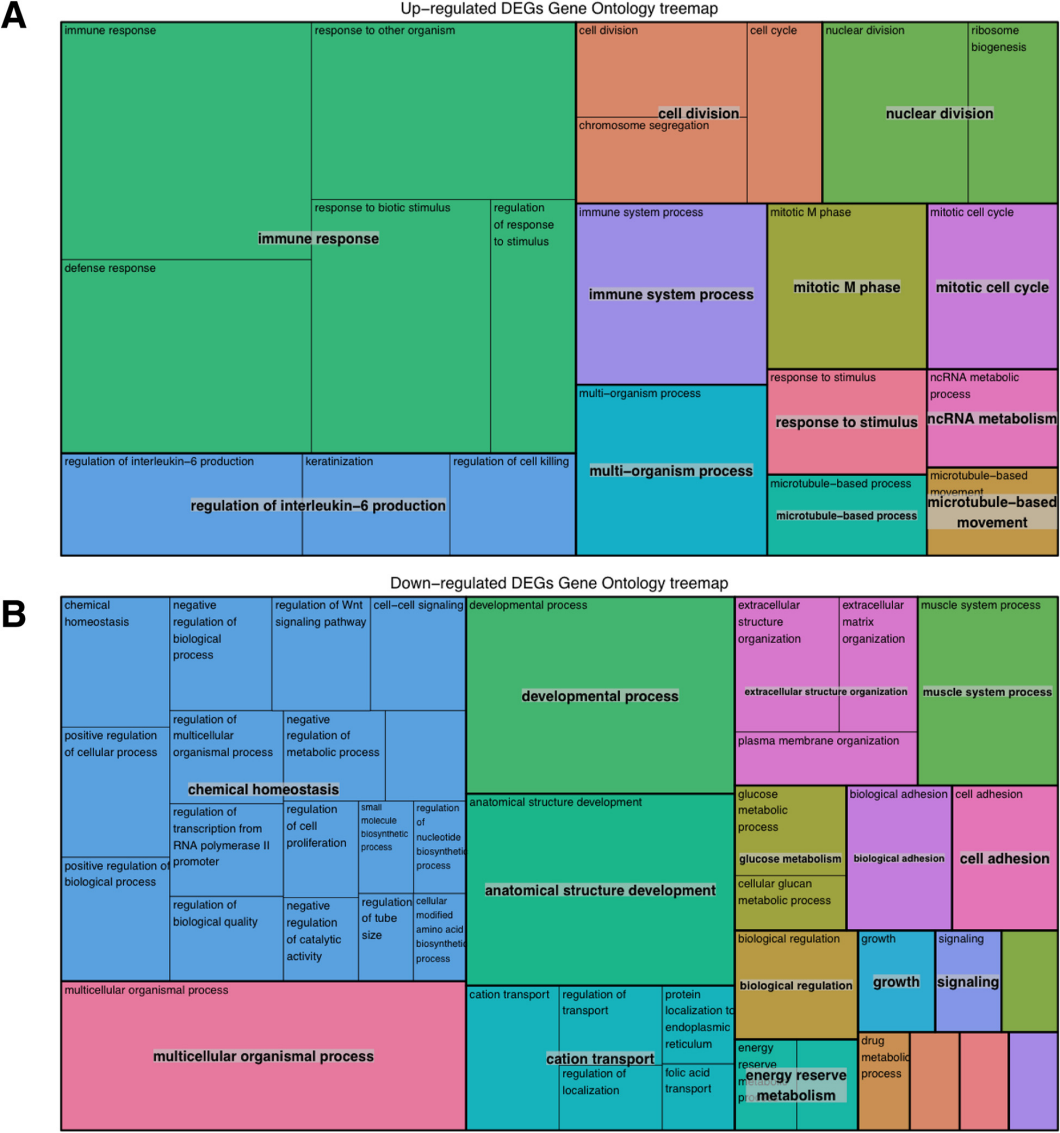
Gene ontology analysis of the up-regulated and down-regulated genes. Gene ontology analysis of the up-regulated and down-regulated genes was conducted using BiNGO. The ontology terms and associated corrected p values were passed to REVIGO which performed summarization by removal of redundant GO terms and was used to generate treemaps of the functional annotations associated with (**a**) the up-regulated and (**b**) the down-regulated genes

In order to investigate the functional associations of the differentially expressed genes, we used the BiNGO tool available in Cytoscape to analyse all up-regulated and down-regulated genes for enrichment of biological processes. We used REVIGO to visualize the results as treemaps (Fig. 2a, b). We see different overarching functional themes emerging in the up-regulated and down-regulated genes. The up-regulated genes are highly enriched for terms involving the cell cycle, immunity and response to stimulus. The down-regulated genes are highly enriched for structural development/morphogenesis and chemical homeostasis. The ontology terms associated with down-regulated genes are also enriched for signalling, ion transport/homeostasis, and cell adhesion. Full details of the gene ontology analysis can be found in Additional file 2: Table S2.

We carried out gene set enrichment analysis (GSEA) using the GSEAPreranked tool to identify gene sets incorporating biological processes and Kegg pathways. This produced results generally consistent with the BiNGO analysis. RNA processing and Cell cycle processes were associated with the up-regulated genes while anatomical structure development and system development were enriched in the down-regulated genes. Enriched KEGG pathways included those involved in RNA processing and cell cycle as well as signalling pathways such as immune system signalling and development signalling pathways such as the hedgehog and Wnt pathways. The gene sets with the highest normalized enrichment scores (NES), are presented in Table 4, the full GSEA results can be found in Additional file 3 :Table S3.

**Table 4:** GSEA analysis of biological process and Kegg pathways most highly ranked gene sets

GSEA Biological process gene set (Up-regulated genes)	NES^a^	GSEA Biological process gene set (Down-regulated genes)	NES^a^
Rna_Processing	4.62	Anatomical Structure Development	−4.05
Cell Cycle Process	4.49	System Process	−3.98
Cell Cycle Go0007049	4.19	System Development	−3.72
Cell Cycle Phase	4.04	Organ Development	−3.69
M Phase	3.97	Multicellular Organismal Development	−3.39
Mitotic Cell Cycle	3.94	Cell Cell Signaling	−3.19
M Phase Of Mitotic Cel Cycle	3.81	Muscle Development	−3.18
Rna Metabolic Process	3.76	Skeletal Development	−3.15
Mrna Metabolic Process	3.66	Anatomical Structure Morphogenesis	−3.04
Dna_Metabolic_Process	3.61	Synaptic Transmission	−2.96
Mitosis	3.60	Organ Morphogenesis	−2.92
Mrna Processing Go0006397	3.39	Striated Muscle Contraction Go 0006941	−2.84
Response To Dna Damage Stimulus	3.38	Ion Transport	−2.75
Dna Repair	3.37	Transmission Of Nerve Impulse	−2.70
Chromosome Organization And Biogenesis	3.18	Amine Metabolic Process	−2.68
Ribonucleoprotein Complex Biogenesis And Assembly	3.14	Nervous System Development	−2.66
Intracellular Transport	2.99	Neurological System Process	−2.62
Translation	2.99	Generation Of Precursor Metabolites And Energy	−2.50
Rna_Splicing	2.97	Regulation Of Growth	−2.44
Nucleocytoplasmic Transport	2.96	Regulation Of Cell Growth	−2.41
Nuclear Transport	2.95	Cation Transport	−2.38
Response To Endogenous Stimulus	2.95	Nitrogen Compound Metabolic Process	−2.36
Establishment Of Cellular Localization	2.89	Metal Ion Transport	−2.29
Cell Cycle Checkpoint Go 0000075	2.89	Calcium Independent Cell Cell Adhesion	−2.26
Cellular Localization	2.85	Transforming Growth Factor Beta Receptor Signaling Pathway	−2.26
GSEA Kegg Pathways gene set	NES	GSEA Kegg Pathways gene set	NES
Kegg Spliceosome	4.06	Kegg Parkinsons Disease	−4.56
Kegg Ribosome	3.93	Kegg Oxidative Phosphorylation	−4.28
Kegg Cytosolic Dna Sensing Pathway	3.46	Kegg Alzheimers Disease	−4.07
Kegg Nod Like Receptor Signaling Pathway	3.45	Kegg Ecm Receptor Interaction	−3.73
Kegg Nucleotide Excision Repair	3.07	Kegg Basal Cell Carcinoma	−3.71
Kegg Rig I Like Receptor Signaling Pathway	2.85	Kegg Huntingtons Disease	−3.67
Kegg T Cell Receptor Signaling Pathway	2.83	Kegg Cardiac Muscle Contraction	−3.18
Kegg Cell Cycle	2.78	Kegg Melanogenesis	−3.12
Kegg Proteasome	2.74	Kegg Hedgehog Signaling Pathway	−3.07
Kegg Rna Degradation	2.68	Kegg Neuroactive Ligand Receptor Interaction	−2.85
Kegg Toll Like Receptor Signaling Pathway	2.60	Kegg Dilated Cardiomyopathy	−2.74
Kegg Aminoacyl Trna Biosynthesis	2.57	Kegg Glycosaminoglycan Biosynthesis Keratan Sulfate	−2.56
Kegg Antigen Processing And Presentation	2.38	Kegg Hypertrophic Cardiomyopathy Hcm	−2.36
Kegg Ubiquitin Mediated Proteolysis	2.35	Kegg Focal Adhesion	−2.33
Kegg Primary Immunodeficiency	2.33	Kegg Calcium Signaling Pathway	−2.28
Kegg Dna Replication	2.33	Kegg Arrhythmogenic Right Ventricular Cardiomyopathy Arvc	−2.27
Kegg Non Homologous End Joining	2.28	Kegg Ppar Signaling Pathway	−2.18
Kegg Homologous Recombination	2.15	Kegg Glycosphingolipid Biosynthesis Ganglio Series	−2.02
Kegg Basal Transcription Factors	2.15	Kegg Peroxisome	−2.01
Kegg Pyrimidine Metabolism	2.15	Kegg Glycosphingolipid Biosynthesis Lacto And Neolacto Series	−2.00
Kegg Epithelial Cell Signaling In Helicobacter Pylori Infection	2.12	Kegg Glycosaminoglycan Biosynthesis Chondroitin Sulfate	−1.98
Kegg Apoptosis	2.06	Kegg Vascular Smooth Muscle Contraction	−1.96
Kegg Mismatch Repair	2.01	Kegg Other Glycan Degradation	−1.89
Kegg One Carbon Pool By Folate	1.88	Kegg Wnt Signaling Pathway	−1.84

^a^NES: Normalised enrichment score calculated using the GSEApreranked tool

### Signalling pathways are dysregulated in *Gtf2ird1* knockout tissue

**Fig. 3:**
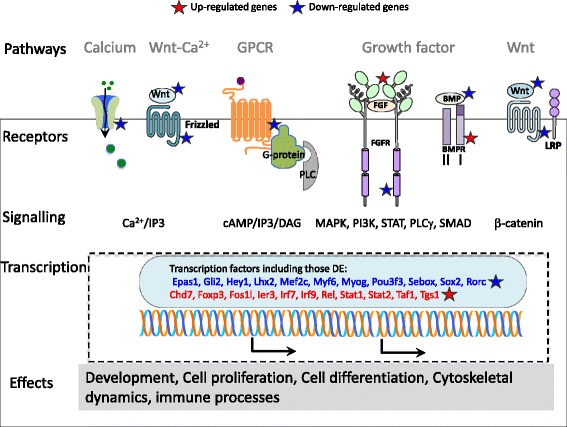
Dysregulated signalling pathways in the absence of *Gtf2ird1*. Schematic diagram indicating signalling pathways with dysregulated gene expression. Lists of differentially expressed genes appearing in the signaling pathways are included in Additional file 4: Tables S1, S3, S4, S7

Our gene ontology analysis and gene set enrichment analysis points to the involvement of multiple signalling pathways within the broad spectrum of gene dysregulation reported in this study. Differentially expressed genes involved in these signalling pathways are detailed in Additional file 4: Table S4 (Tables S1–S7). A schematic of the primary dysregulated signalling pathways is presented in Fig. 3. We see altered expression of growth factors (Additional file 4: Table S1), stimulating cytokines (Additional file 4: Table S2), Wnt signalling molecules (Additional file 4: Table S3), calcium signalling (Additional file 4: Table S4), cell cycle genes (Additional file 4: Table S5), hedgehog signalling (Additional file 4: Table S6) and G protein-coupled receptor signalling (Additional file 4: Table S7). Notably, we see significant up-regulation of genes which stimulate growth factor signalling such as *Ngf* (nerve growth factor) and *Fgf1* (fibroblast growth factor 1) as well as genes that enhance growth factor signalling such as *Fgfbp1* (FGF-binding protein)[22]. While *Ngf,* and *Fgf1* have increased expression in the KO, the genes encoding their respective receptors, *Ngfr* and *Fgfr1* have decreased expression.

We see down-regulation of members of the transforming growth factor family, *Tgfb2* and the bone morphogenetic proteins, *Bmp4* and *Bmp6*. Stimulation of the canonical pathway via TGFβ or the BMPs typically results in phosphorylation and activation of the SMAD transcription factors [23]. We find no significant change in expression of the activating SMADs. Down-regulation of *Tgfb2*, which is an inhibitor of cellular proliferation, is consistent with the other evidence of increased cell division and proliferation seen in the KO phenotype [6].

The Wnt genes*, Wnt7b, Wnt10b*, and *Wnt11* are all down-regulated in the KO. The most significantly down-regulated of these is *Wnt7b*, which is active in the canonical Wnt pathway. This is followed by *Wnt11*, which activates the non-canonical Wnt-calcium pathway that affects cytoskeletal dynamics and cell adhesion [24, 25] and *Wnt10b*, known to promote the differentiation of skin epithelial cells and the development of hair follicles [26]. More generally, we find that a number of genes involved in calcium signalling are differentially expressed in the KO, as listed in Additional file 4: Table S4.

Increased growth factor signalling might be expected to result in increased cellular proliferation. We note that several of the up-regulated genes are involved in creating the cytoarchitecture of microtubules required for cell division during the mitotic phase of the cell cycle. For example, we see up-regulation of genes involved in cytokinesis or spindle formation and stabilization, such as *Prc1, Anln, Aspm, Cenpe, Spdl1, Kif11, and Spag5*. We also see increased expression of genes involved in chromatin condensation and the correct segregation of chromosomes during cell division, such as *Sgol1, Smc1a, Smc2, Smc3 and Smc4*. (Additional file 4, Table S5).

### Genes known to be regulated by GTF2IRD1 and other known interaction partners of GTF2IRD1

The large number of differentially expressed genes identified in this study contrasts with the small number of direct interaction partners of GTF2IRD1 that are currently annotated, in the MetaCore and BioGRID databases (http://thebiogrid.org). The targets listed in the MetaCore database are primarily genes thought to be transcriptionally regulated by GTF2IRD1. The interactions listed in the BioGRID database are protein targets identified by Affinity-Capture and Two-hybrid methods. Our search of the MetaCore database produced 16 interaction partners and our search of the BioGRID databases produced 13 interaction partners. Our search results can be found in Additional file 5: Table S5. We recently reported work involving an unbiased screening strategy for interaction partners of GTF2IRD1 to rectify this shortcoming [27]. Our study identified 38 novel interaction partners that are mostly involved in chromatin modification and transcriptional regulation as well as proteins associated with the primary cilium [27].

We have looked at whether any of the identified GTF2IRD1 interaction partners are differentially expressed in this RNA-Seq study, since the genes that encode these proteins may belong to a regulatory gene network that is active at the transcriptional and post-translational levels. Among the genes identified in the MetaCore™ and BioGRID databases we find decreased expression of two genes that have been implicated in transcriptional activation by GTF2IRD1, *Ccnd3* (Cyclin D3) expression is decreased by 30 % (FDR = 0.065) and *Tgfb2* (Transforming growth factor beta 2) decreased by 45 % (FDR = 0.002). *Ccnd3* plays an important role in cell cycle progression and in phosphorylation of RB1. In addition to its interaction with RB1 and GTF2IRD1, CCND3 also affects other transcription factors and may have a repressive effect (for example on *Runx1* [28] and the androgen receptor [29]) or may have an activating effect such as for *Pparg* [30], *Rara* [31] and *Vdr* [32]. TGFβ2 plays a role in the process that leads to the phosphorylation of SMAD, its translocation to the nucleus and ultimately transcriptional activation [33].

Our RNA-Seq results show a significant increase in expression in the Gtf2ird1 KO of *Kpna2, Atf7ip, Parpbp* which code for proteins identified by Carmona-Mora et al. (2015)*.* We note that *Trip11* and *Alms1* are also increased but at an FDR of 0.061 and 0.065 respectively. It is interesting that each of these genes has been reported as having an association with the cell cycle. *Atf7ip* encodes a transcriptional coactivator or corepressor involved in cell cycle arrest [34, 35]. *Parpbp* plays a role in chromatin modulation, DNA repair and cell cycle progression [36]. KPNA2 participates in nucleocytoplasmic transport and is associated with the cell cycle and DNA repair [37]. ALMS1 has a role in the cell cycle through its involvement in formation and maintenance of cilia. TRIP11 is involved in the microtubule network and in transporting proteins to the ciliary membrane [38, 39].

### Validation of RNA-Seq results with RTqPCR

**Fig. 4:**
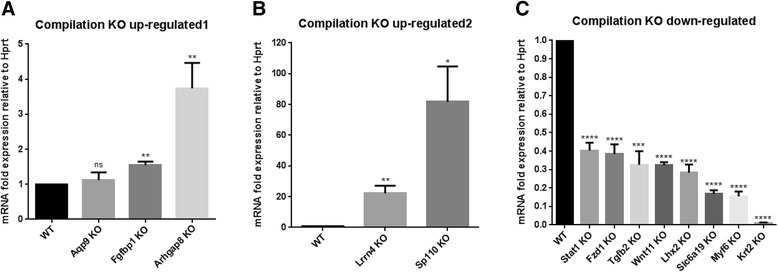
RTqPCR validation of dysregulation in 13 genes identified by RNA-Seq analysis. Fold expression change relative to the mean expression level of the wild type mice, which is set at 1 in all cases. Error bars represent SEM (*p value ≤0.05, ** *p* value ≤0.002, *** *p* value ≤ 0.001, **** *p* value ≤ 0.0001). **a** Expression of *Fgfbp1* and *Arhgap8* was elevated in KO when normalized to the WT. No statistical difference was found in *Aqp9*. **b** *Lrrn4* and *Sp110* were elevated in KO when normalized to the WT. **c** Expression of *Stat1*, *Fzd1*, *Tgfb2*, *Wnt11*, *Lhx2*, *Slc6a19*, *Myf6* and *Krt2* was decreased in KO when normalized to the WT

In order to verify the RNA-Seq results, we conducted RTqPCR analysis. CDNA was prepared from four lip tissue RNA samples per genotype. Transcript levels for the genes analysed were measured in comparison with the housekeeping gene *Hprt* (hypoxanthine phosphoribosyltransferase 1) as an internal reference standard, using the 2^- ΔΔCT^ method [40]. Thirteen genes found to be differentially expressed in the RNA-Seq analysis were tested: *Lrnn4*, *Sp110*, *Aqp9*, *Arhgap8*, *Lhx2*, *Stat1*, *Wnt11*, *Fzd1*, *Tgfb2*, *Fgfbp1*, *Slc6a19*, *Myf6* and *Krt2*. The genes tested are involved in different functional categories discussed in this paper, rather than being focused on a single pathway in an attempt to provide a broad confirmation of the RNA-Seq experiment. The results of this analysis are presented in Fig. 4 (a–c). We observed a statistically significant increase in *Fgfbp1, Arhgap8, Lrnn4, Sp110* (Fig. 4a, b) and a statistically significant decrease in *Lhx2, Stat1, Fzd1, Tgfb2, Wnt11, Slc6a19, Myf6 and Krt2* (Fig. 4c), in agreement with the RNA-Seq analysis. Comparative expression levels were calculated and the mean transcript level found in the wild type samples was set at a value of 1 in order to plot all genes on the same graph (Fig. 4a–c).

All of these genes tested, with the exception of *Aqp9* which had high variation in the KO samples, were found to be significantly differentially expressed in conformity with our RNA-Seq results.

## Discussion

In this work, we analysed the transcriptome of lip tissue from a *Gtf2ird1* knockout mouse line. This line was created to better understand the effect of loss of the human orthologue of this gene in WBS, which is a neurodevelopmental disorder caused by the hemizygous deletion of *GTF2IRD1* and other genes within the Williams-Beuren syndrome critical region of 7q11.23. Prior studies point to *GTF2IRD1* affecting craniofacial development, cognition and behaviour [3, 5, 6, 8].

Using RNA-Seq, we have identified a large number of up-regulated and down-regulated genes, revealing that the inactivation of *Gtf2ird1* has widespread effects. Our functional analysis indicates that these changes in gene expression may impact structural/morphological development of tissue, cellular differentiation, cellular proliferation and the immune response and that these processes may be mediated through aberrant signalling. Key molecular factors in development are transcription factors, signalling molecules and cell adhesion molecules and in this study we detect gene dysregulation in all three categories. We note overlap between the gene ontology terms highlighted in this study and those emerging from earlier microarray studies, indicating involvement of signalling, cell cycle and immune response in WBS gene dysregulation [17, 41].

Although GTF2IRD1 is known to regulate gene transcription [16, 42, 43], a relatively small number of direct targets of GTF2IRD1 have been identified to date. GTF2IRD1 has been shown to complex with the transcriptional repressor RB1, leading to the suggestion that it may be a general transcription factor, regulated through its association with RB1 and involved in cell-cycle progression [44]. RB1 controls cell cycle progression by interacting with the E2F family, preventing the cell from progressing from G1 to S phase and by attracting histone deacetylases (HDACs) to chromatin, thus suppressing DNA synthesis. It has also been demonstrated that GTF2IRD1 associates with HDAC3 and to a lesser extent with HDAC1 [45]. It has been suggested that the transcriptional activity of the GTF2I family may be modulated by the HDAC proteins during development, with this regulation being further refined by the activity of PIAS2 (an E3-type small ubiquitin-like modifier [SUMO] ligase) which associates with HDAC3 [45]. Interestingly, we do see a strong signature of dysregulation in the cell cycle with up-regulation of many genes involved in cell division and proliferation. This observation, in the absence of any change in *Rb1* expression, could be explained by a change in the post-translational modification of RB1. In this study, we see a decrease in *Ccnd3* (FDR = 0.065) a gene that encodes a protein involved in the phosphorylation of RB1. An alternative or ancilliary explanation is that GTF2IRD1 may normally assist RB1 to find or bind to its targets and that its absence results in dysregulation of genes downstream of RB1, observed as increased cellular proliferation.

If GTF2IRD1 plays a direct role in cell proliferation through these mechanisms, one must ask why excess proliferation has not been seen in other tissues of the knockout mice where *Gtf2ird1* is normally expressed. One possible explanation is that, unlike skin, these other sites undergo terminal differentiation and are not part of a system that undergoes continuous renewal through the activity of stem/progenitor cells and are therefore not in a position to respond to the change in GTF2IRD1 status in this way.

GTF2IRD1 has previously been associated with transcriptional repression [13, 14] which predicts that the majority of direct targets would show an increase in expression in the KO. It is noteworthy that the most statistically significant differentially expressed genes identified in this study are within the up-regulated group.

Our analysis points to dysregulation of a number of transcription factors involved in tissue development. We have drawn attention to one of these factors *Lhx2*, which may be especially relevant to WBS as it is active in tissue development and in neurological processes. LHX2 regulates hair follicle development [46] and skin repair and has been described as a central link in the genetic networks that coordinate multiple signalling pathways controlling organ development and cell fate determination [47]. LHX2 also regulates brain development [20] and is an activator of SOBP [47] which has been observed to have strikingly specific expression in the limbic system, with disruption leading to abnormal cognition and intellectual disability [48]. The putative effects of LHX2 seem highly relevant to WBS. We have therefore validated change in expression of *Lhx2* with qPCR. In addition, our RNA-Seq analysis showed corresponding expression changes in many genes normally regulated by LHX2.

The dysregulation of genes that encode signalling molecules, which ultimately impact on transcriptional regulation, would also be predicted to contribute to the large number of differentially expressed genes detected in this study. This includes factors like CCND3, which has a repressive effect on *Runx1* [28] and the androgen receptor [29] and an activating effect on *Pparg* [30], *Rara* [31], *Vdr* [32] and TGFβ2 through its actions in the SMAD pathway.

Histological analysis has revealed dysfunctions in cellular proliferation and differentiation in the skin overlying the lips in *Gtf2ird1* knockout mice [6]*.* It is possible that these dysfunctions stem from dysregulation of signalling pathways. We have found altered expression of genes involved in multiple signalling pathways including growth factor signalling, Wnt, BMP and hedgehog signalling. These pathways act in a highly coordinated manner during the development of many tissues, although the mechanisms of cross-talk are still areas of active research [49–51].

Several members of the fibroblast growth factor (FGF) family are dysregulated (Additional file 4: Table S1). The FGFs regulate development by orchestrating mesoderm patterning in the early embryo and then guiding organ development by regulation of cell proliferation, differentiation and survival [52]. Genes from this family are expressed in keratinocytes throughout all layers of the epidermis where they stimulate skin activity including induction of the induction of keratinocyte proliferation [22].

Members of the FGF family instigate signalling though the canonical mitogen-activated protein (MAP) kinase pathway, the STAT pathway, the PI3 kinase/AKT pathway and the PLCγ pathway [53]. The MAP kinase pathway is involved in cell growth and differentiation, the PI3K pathway is implicated in cell survival and polarity control and the PLCγ pathway may be necessary for cell adhesion [54]. Therefore, the fibroblast growth factors have a wide impact on biological activities that are of direct relevance to the epidermal phenotype seen in the KO [52, 54].

Fibroblast growth factor signalling also plays a critical role in brain development. Gene inactivation studies have shown that the receptors FGFR1 and FGFR2 are necessary for brain development [55] and proper formation of the medial prefrontal cortex and its connections with the limbic circuits [56]. As well as seeing altered expression in *Fgf1* and *Fgfr1,* we also see a 46 % decrease in *Fgfbp3,* a gene that has been associated with the regulation of anxiety [57]. It is plausible that some of the gene expression changes seen in this study also occur when GTF2IRD1 levels are reduced in the brain, leading to dysregulation of FGF signalling and its functions in limbic system control. This could explain some of the behavioural abnormalities that are characteristic of WBS.

The interplay between FGF signalling and other signalling pathways is complex and will differ from tissue to tissue. However, it is known that the signalling pathways stimulated by the FGFs may also be stimulated by TGFβ, BMPs (the bone morphogenetic proteins) and Wnt ligands [58]. In addition to changes in FGF signalling genes, we report decreased expression of *Tgfb2*, BMPs and Wnt signalling genes in the KO.

Overall, it appears that signalling through these ligands is decreased in the absence of *Gtf2ird1*. We therefore suggest that biological outcomes such as TGFβ-induced epithelial-to-mesenchymal (EMT) transition and TGFβ-induced axonal outgrowth may be affected. The TGFβ proteins generally have an anti-proliferative effect in epithelial cells as well as regulating the immune response. Amelioration of this anti-proliferative action is consistent with the hyperproliferation of epidermal cells seen in the *Gtf2ird1* KO phenotype [6].

The Wnt signalling pathway is also involved in the same key biological processes of cellular proliferation, differentiation, adhesion and survival in a range of tissues, and plays an important role in the nervous system [49]. The Wnt proteins mediate transduction in three major pathways, the canonical Wnt/β-catenin pathway, the planar cell polarity pathway and the Wnt-calcium pathway [24]. In each of these pathways, the Wnt ligand binds to its cognate receptor, Frizzled. In this study, we find that genes encoding four of the Frizzled receptors (*Fzd1, Fzd2, Fzd5* and *Fzd9*) are significantly down-regulated in the KO. The most significantly down-regulated Wnt gene is *Wnt-7b*, which is active in the canonical Wnt pathway. This is followed by *Wnt-11*, which activates the non-canonical Wnt-calcium pathway that affects cytoskeletal dynamics and cell adhesion [24, 25], and *Wnt-10b,* known to promote the differentiation of skin epithelial cells and development of hair follicles [26]. We find decreased expression of genes in the Wnt-calcium pathway, as well as in calcium ion homeostasis. This is interesting as calcium signalling pathways are known to be key in keratinocyte proliferation, migration and differentiation [59], inflammation [60] and the nervous system [61], all relevant to the WBS phenotype.

Patients with WBS display a number of cognitive and behavioural abnormalities. In this study, we have observed that the differentially expressed genes are enriched with gene ontology terms relevant to neural processes. FGF signalling, Wnt signalling, and other forms of calcium signalling are key in a number of neural processes including neurogenesis, axon outgrowth and guidance, neuronal polarity, dendrite development, dendritic spine differentiation and synapse formation and maintenance [62]. It is interesting that the calcium regulated oxytocin receptor is down-regulated in the KO. Dysregulation of oxytocin signalling is known to affect human behaviours involving trust and bonding [63], which may be particularly relevant to WBS [64].

It must be borne in mind that we have only tested lip tissue in this study. However, it is plausible that the mechanisms of GTF2IRD1 regulation that are disrupted here also operate in other tissue types including the brain. Microarray analysis of *Gtf2ird1* knockout brain tissue has so far failed to identify significant levels of transcriptional dysregulation [15]. However, the brain is a particularly difficult system for such investigations due to the complexity and cellular diversity; any alterations that occur within neuronal sub-populations are very hard to isolate and observe. It is useful, therefore, to generate hypotheses in proxy tissue systems, such as the lip tissue used here, and then apply these hypotheses in more specific ways to the brain.

In summary, we see widespread gene dysregulation in the lip tissue of mice in which *Gtf2ird1* has been inactivated. We suggest that the loss of GTF2IRD1 has a direct effect on the expression of some genes, as well as altering the transcriptional control of other genes through the disruption of downstream transcription factors and signalling pathways. The combined effect leads to widespread gene dysregulation affecting multiple developmental mechanisms.

## Conclusions

We have demonstrated that RNA-Seq is a powerful method for investigating global transciptomic changes resulting from *Gtf2ird1* inactivation. This analysis directs our attention to mechanisms at play in neurodevelopmental conditions such as WBS, which involves the hemizygous deletion of *GTF2IRD1* as well as other genes. Overall, we see widespread changes in expression of genes involved in tissue development and functional maintenance. This is evident in the samples of epidermal tissue analysed in this experiment but also has implications in other tissue such as the brain. Several of the genes found to be dysregulated play fundamental roles in a range of tissues including the brain and may be highly relevant to WBS. While the genes found to be dysregulated help to elucidate the processes involved in WBS, we are some way from understanding the mechanism by which GTF2IRD1 brings about these changes in gene expression. It is possible that GTF2IRD1 engages in multiple interactions with other nuclear factors and the complexes that are formed become located at a variety of genomic loci in order to regulate transcription. If the primary targets of GTF2IRD1 are other transcription factors and genes involved in signalling pathways, this could account for the broad gene dysregulation seen in this study. A clearer understanding of GTF2IRD1 function should, therefore, emerge from studies aimed at identifying protein-protein interactions, transcriptomics in a variety of tissues and assays designed to identify direct gene targets.

## Methods

### Animals

*Gtf2ird1*^*tm1Hrd*^ mice, referred to as knockout (KO) mice, were described previously [6]. The mutation has been maintained on a C57BL/6 J background for greater than 20 generations and the experiments involved use of mice on this background. All experimental procedures were approved by the Animal Care and Ethics Committee at UNSW Australia. The mice used in the RNA-Seq and qPCR experiments were all adult females (2–6 months).

### Tissue preparation

After mouse euthanasia by cervical dislocation, lip tissue from KO and WT mice were carefully cleaned with 75 % ethanol and DEPC-treated water. The top lip was then dissected and immediately immersed in 2 mL of cold TRI-reagent (Sigma). Lip tissues were homogenised until fully dissociated (approximately 30 s) using a T10 Ultra-Turrax homogeniser (Themo-Fisher Scientific) for subsequent RNA extraction. Tail biopsies were collected to confirm that the genotyping results established at 3 weeks old were correct.

### Total RNA extraction

RNA was extracted from the dissected mouse tissues using TRI-reagent (Sigma), following the manufacturer’s instructions, which included a chloroform phase separation and ethanol precipitation. RNA was resuspended in 70 μL of RNase-free water. RNA samples were assessed for quantity and quality using a NanoDrop UV spectrophotometer (Thermo Fisher Scientific Inc), conventional RNA electrophoresis and using a Bioanalyser (Agilent Technology Inc). All RNA integrity numbers (RIN) for the analysed samples ranged from 9 to 9.5

### Transcriptome sequencing

RNA extracted from 3 WT mice and 3 KO mice was used to prepare six mRNA libraries following the standard Illumina protocol. The six RNA-seq libraries were sequenced on the Illumina HiSeq2000 platform at the Ramaciotti Centre for Genomics UNSW, to produce over 60 million, 100 nucleotide paired-end reads per sample (Reads 1 and 2).

### Mapping RNA-Seq reads

The reads were mapped to the Ensembl *Mus musculus* genome (GRCm38) provided by Illumina iGenomes (downloaded from cufflinks.cbcb.umd.edu/igenomes.html). Mapping was performed with Tophat2 (v 2.0.8) [65] calling Bowtie2 (v 2.1.0) [66] using the default settings.

HTSeq-count (Python package HTSeq, python v 2.7.3) was used to generate counts of reads uniquely mapped to annotated genes using the GRCm38 annotation gtf file. HTSeq-count produced over 20 M uniquely aligned reads per sample (WT1 = 27811674, WT2 = 22050557, WT3 = 23415253, KO1 = 20164081, KO2 = 29491767, KO3 = 22204515). 26814 genomic features were counted with at least one read.

### Differential gene expression analysis

We performed differential expression analysis using the count based method, edgeR (v 3.8.6) [18] and we confirmed these results using DESeq2 (v 1.6.3) [19], both tools are available as Bioconductor packages. Tables of raw counts generated using HTSeq-count (described above) were used as input in both analyses.

In the edgeR analysis, low count transcripts were excluded and only those genes with at least 1 count per million (cpm) in at least 3 samples were used for analysis. A normalization factor was calculated using the trimmed mean of M values (TMM) method [67] and the dispersion parameter for each gene was estimated using the quantile-adjusted conditional maximum likelihood (qCML) method, appropriated for experiments with a single factor. The functions estimateCommonDisp() and estimateTagwiseDisp() were used to estimate dispersion. Following this we tested for differential expression using the exact test based on qCML methods. The Benjamini-Hochberg correction was used with a false discovery cut-off of 0.1.

DESeq2 uses a generalised linear model (GLM) to assess differential expression. Dispersions were estimated using a Cox-Reid adjusted profile likelihood and the Wald test for significance of GLM was used. Automatic filtering is incorporated to exclude low abundance genes in the testing process.

### Functional analysis

We used the BiNGO plug-in to Cytoscape [68] to investigate the functional associations of genes found to be either down-regulated or up-regulated. As background we used the set of 14,526 genes used in our differential expression analysis. Of these, 13,376 genes were used in the gene ontology analysis. The Gene Ontology (GO) terms in the category Biological Process were tested for overrepresentation using the hypergeometric test and p values were corrected using the Benjamini & Hochberg FDR correction. Enriched GO were selected using a corrected p value of 0.01.

We used the REVIGO tool [69] to visualize the gene ontology terms associated with the differentially expressed genes.

We used the GseaPrePranked tool to indentify the enriched gene sets in c5.bp.v5.1.symbols.gmt of MSigDB (biological process) and in c2.cp.kegg.v5.1.symbols.gmt of MSigDB (Kegg pathways) with a permutation of 1000. An FDR q-value were of 0.05 was adopted [70].

We used MetaCore™ from Thomson Reuters to interrogate the list of differentially expressed genes for associated transcription factor factors. This allowed us to identify 78 transcription factors which are differentially expressed. MetaCore™ comprises a suite of software and an extensive database, which contains manually curated information on proteins, genes, complexes, metabolites, RNA and DNA and their interactions gleaned from the published literature. We also used MetaCore™ to find interaction partners of genes of interest.

We used BiNGO to obtain gene ontology terms associated with the differentially expressed transcription factors.

### Validation with RTqPCR

#### Reverse transcription

First-strand cDNA synthesis was carried out using the SuperScript® III Reverse Transcriptase (Invitrogen™), with Random Hexamer Primer (Thermo Scientific™) using 1 μg of total RNA as template, according to the manufacturer’s instructions.

### Quantitative real time-PCR

CDNA was prepared from four lip tissue RNA samples per genotype (4 adult WT mice and 4 adult KO mice).

Small samples of the cDNA (1–2 % of the total) were used as a template for quantitative PCR (qPCR) using the Fast SYBR® Green Master Mix (Applied Biosystems™). Reactions were set up to a total volume of 10 μL according to the product protocol and performed on the Stratagene MX3005P qPCR system (Agilent Technologies). Each reaction was set up in triplicate for the target gene under test. Triplicate reactions were also set up with an identical amount of template using primers designed against mouse *Hprt* (hypoxanthine phosphoribosyltransferase 1) as a housekeeping gene reference standard (see Additional file 6 for primer sequences).

For all RTqPCR assays, the efficiency of the different primer sets was tested by establishing a standard curve using serial dilutions of a cDNA pool made by combining samples of all the templates used in each experiment. MxPro QPCR Software was used to analyse the dissociation and amplification curves of every experiment and to obtain the threshold cycle values (Ct). Data were then analysed using Microsoft Excel for quantitation of target gene relative to the reference standard using the 2^- ΔΔCT^ method [40]. *T*-test analyses were performed on the 2^- ΔΔCT^ values for the two groups of samples.

## Additional files

Additional file 1: S1. Differentially expressed genes found by edgeR. This file contains the analysis results for all genes tested by edgeR. (XLSX 1794 kb) Additional file 2: Table S2. Gene ontology analysis performed with the BiNGO plug-in to Cytoscape. The lists of up-regulated and down-regulated genes were analysed for enrichment of gene ontology terms using BiNGO. The results are presented in this file. The results of gene ontology analysis for the DE transcription factors are also in this file. (XLSX 154 kb) Additional file 3: Table S3. Gene set enrichment analysis using the GSEApreranked tool. The full results of the GSEA analysis for biological process and Kegg pathways are in this file. (XLSX 96 kb) Additional file 4: Table S4. (Tables S1–S7) Categorisation of Subsets of differentially expressed genes. This file includes tables of genes found to be differentially expressed which are involved in growth factor signalling (Table S1), cytokine signalling (Table S2), Wnt signalling (Table S3), Calcium signalling (Table S4), Cell cycle (Table S5), Hedgehog signalling (Table S6) and G protein-coupled signalling (Table S7). (XLSX 89 kb) Additional file 5: Table S5. Searches for GTF2IRD1 interaction partners in the BioGRID and MetaCore databases. (XLSX 51 kb) Additional file 6: Table S6. Primers used in RTqPCR validation. (XLSX 10 kb)
